# Transcriptome-wide modulation by *Sargassum vulgare* and *Acanthophora spicifera* extracts results in a prime-triggered plant signalling cascade in tomato and sweet pepper

**DOI:** 10.1093/aobpla/plac046

**Published:** 2022-10-06

**Authors:** Omar Ali, Adesh Ramsubhag, Jayaraj Jayaraman

**Affiliations:** Department of Life Sciences, Faculty of Science and Technology, The University of the West Indies, St. Augustine TTO, 00000, Trinidad and Tobago; Department of Life Sciences, Faculty of Science and Technology, The University of the West Indies, St. Augustine TTO, 00000, Trinidad and Tobago; Department of Life Sciences, Faculty of Science and Technology, The University of the West Indies, St. Augustine TTO, 00000, Trinidad and Tobago

**Keywords:** *Acanthophora spicifera*, biostimulant: macroalgae, priming, *Sargassum vulgare*, seaweed extracts, sweet pepper, tomato, transcriptome

## Abstract

Seaweed extracts (SWEs) are becoming integrated into crop production systems due to their multiple beneficial effects including growth promotion and induction of defence mechanisms. However, the comprehensive molecular mechanisms of these effects are yet to be elucidated. The current study investigated the transcriptomic changes induced by SWEs derived from *Sargassum vulgare* and *Acanthophora spicifera* on tomato and sweet pepper plants. Tomato and sweet pepper plants were subjected to foliar treatment with alkaline extracts prepared from the above seaweeds. Transcriptome changes in the plants were assessed 72 h after treatments using RNA sequencing. The treated plants were also analysed for defence enzyme activities, nutrient composition and phytohormonal profiles. The results showed the significant enrichment of genes associated with several growth and defence processes including photosynthesis, carbon and nitrogen metabolism, plant hormone signal transduction, plant–pathogen interaction, secondary metabolite metabolism, MAPK signalling and amino acid biosynthesis. Activities of defence enzymes were also significantly increased in SWE-treated plants. Plant nutrient profiling showed significant increases in calcium, potassium, nitrogen, sulphur, boron, copper, iron, manganese, zinc and phosphorous levels in SWE-treated plants. Furthermore, the levels of auxins, cytokinins and gibberellins were also significantly increased in the treated plants. The severity of bacterial leaf spot and early blight incidence in plants treated with SWE was significantly reduced, in addition to other effects like an increase in chlorophyll content, plant growth, and fruit yield. The results demonstrated the complex effect of *S. vulgare* and *A. spicifera* extracts on the plants’ transcriptome and provided evidence of a strong role of these extracts in increasing plant growth responses while priming the plants against pathogenic attack simultaneously. The current study contributes to the understanding of the molecular mechanisms of SWEs in plants and helps their usage as a viable organic input for sustainable crop production.

## Teaser

Seaweed extracts derived from *Sargassum vulgare*and *Acanthophora spicifera*, when applied as foliar sprays on tomato and sweet pepper plants, were able to evoke several genetic defence and growth responses in a harmonious way through transcriptome modulations. This induced response contributed to the significant decrease in disease levels (both fungal and bacterial) observed in both crop plants as well as simultaneous increased growth inclusive of plant height, chlorophyll content and overall fruit yield.

## Introduction

The establishment of sustainable agricultural practices is at the forefront of today’s ever-changing environment. The reliance and rampant misuse of pesticides in modern crop production systems have resulted in major challenges to global agricultural and environmental sustainability (Battacharyya *et al.* 2015; Ali *et al.* 2021a). Pathogenic resistance, environmental pollution, toxic bioaccumulation of pesticides in produce, pesticide poisoning and high food-importation bills are influenced by the dependency on chemical pesticides, especially in developing nations where their use remains largely unregulated and unmonitored (Aktar *et al.* 2009; Kesavachandran *et al.* 2009). Therefore, newer and innovative methods are urgently needed for establishing sustainable agricultural systems. One such promising method is the incorporation of seaweed-based biostimulants in crop management protocols since a large number of studies have recorded multiple beneficial effects in many economically important crops including tomato, sweet pepper, cucumber, carrot and strawberry (El Boukhari *et al.* 2020; Ali *et al.* 2021a). The application of seaweed extracts (SWEs) has been shown to promote plant growth and yield in greenhouse and field conditions (Ali *et al.* 2019, 2021b) and even in home-garden growing conditions (Rajendran *et al.* 2022). Additional SWEs have also been able to enhance plant resistance towards a myriad of pathogens through the elicitation of various growth and defence signalling networks (Tuchy *et al.* 2013) and even to abiotic stresses (Kasim *et al.* 2015; Xu and Leskovar 2015; Ali *et al.* 2018).

Previous work done utilizing extracts prepared from *Sargassum vulgare* and *Acanthophora spicifera* in tomato and sweet pepper showed that crops treated with the extracts had lower disease severity levels and produced higher yields. Further investigations also concluded that the extracts had no direct antimicrobial activities. Instead, *S. vulgare* and *A. spicifera* extracts were able to elicit defence and growth responses in the two crops via genetic modulation of defence genes. These results showed the upregulation of marker genes including *PR-1a*, *PinII* and *ETR-1*, which are involved in the salicylic acid (SA), jasmonic acid (JA) and ethylene (Et) pathway-mediated defence signalling. Simultaneously, genes involved in the biosynthesis of cytokinins (*IPT*), gibberellins (*Ga2Ox*) and auxins (*IAA*) were also upregulated (Ali *et al.* 2021b). These studies were done using the gold-standard qPCR technique, which, although is very accurate, remains very targeted only to certain known genes of interest. However, RNA-sequencing approaches allow for the documentation of global changes in the transcriptome in response to a treatment, and hence, it is regarded as the ‘catch-all technique’ (Stark *et al.* 2019). The current study therefore utilized next-generation RNA sequencing to capture the global transcriptome changes in tomato and sweet pepper in response to foliar treatment with *S. vulgare* and *A. spicifera* SWEs as a means to elucidate their mechanisms of action. The information gathered from the study will provide insights into the mechanisms of action of SWEs which could therefore be corroborated with the previously demonstrated elicitor activities of SWEs in plants.

## Methodology

### Experimental design

In a greenhouse, healthy 6-week-old tomato (Hybrid-61) and sweet pepper (Amrit) plants were transferred into 1-gallon plastic containers with a 1:1 peat moss and soil combination. The plants were grown at 30°C, 70–85 % relative humidity and 600–1000 μmol photons per m^2^ per second with ~12-h photoperiod. Irrigation was supplied via a drip system with each plant receiving 20 mL water, three times a day (Ali *et al.* 2022).

*Sargassum vulgare* (TRIN 50547) and *A. spicifera* (TRIN 50548) were collected from the north coast of Trinidad, West Indies. Extracts of *S. vulgare* (SV) and *A. spicifera* (AS) were prepared using a 2 % KOH alkaline solution according to Ali *et al.* (2021b). Samples of both extracts were subsequently sent to J.H.G. Analytical Services Limited, Waterford, Ireland, for compositional profiling. The analysis included major hormones, and polysaccharide bioactive analyses including laminarins, fucoidans and alginic acid. Plants were treated with a foliar spray of 0.5 % v/v SWE (AS or SV) (5 mL SWE per litre) at a rate of 15 mL per plant and a water control after 1 week of acclimatization. A fine mist was applied to only allow for foliage cover and to avoid unnecessary drippage to the soil. The plants were sampled for gene expression analysis 72 h after treatment by collecting two fully expanded leaves at the third and fourth nodes from the base of the stem. The samples were instantly transferred to liquid nitrogen and used for RNA extraction. There were 20 plants maintained per treatment, and duplicate plants were sampled for each treatment.

### RNA extraction and Illumina high-throughput sequencing

The Trizol reagent (Invitrogen, USA) was utilized to extract total RNAs from 500 mg of leaf tissue as per the guidelines from the manufacturer. The quantification of RNA was carried out using a Jenway Genova NanoSpec and a denaturing agarose gel electrophoresis was employed to check the integrity of the RNA. The RNAs from two biological replicates were sent for RNA sequencing (Novogene, USA). Agarose gel electrophoresis, a Thermo-Fisher NanoDrop spectrophotometer, and the Agilent 2100 bioanalyser were used to quantify and assess integrity and purity of the extracted RNA. Samples with ≥20 ng μL^−1^ RNA (≥20 μL), OD_260/280_ ≥ 2.0, OD_260/230_ ≥ 2.0 and an RNA integrity number (RIN) ≥ 6.3, with a flat baseline were considered usable for sequencing. The cDNA library was made utilizing the Illumina RNA NEBNext Ultra II kit. The library quality was assessed using Qubit 2.0 (library concentration preliminary test), Agilent 2100 bioanalyser (insert size assessment) and qPCR (to accurately calculate the library’s effective concentration). RNA sequencing was done via the NovaSeq 6000 platform to generate ~20 million paired-end reads for each sample. The experiment’s raw RNA-sequencing fastq data files were deposited in the NCBI’s Short Read Archive (SRA) database under the bioproject PRJNA782171. The sequencing read statistics can be seen in **Supporting Information—Table S1**).

### Sequence quality assessment, mapping and differential expression analysis (reference-based)

The bioinformatic analysis **[see****Supporting Information—Fig. S1****]** was done using the Galaxy Platform (https://galaxyproject.eu/) (Afgan *et al.* 2018). FastQC Version 0.72 was used to examine the quality of the raw sequencing reads (Andrews *et al.* 2015). The reads were filtered using Trimmomatic, Galaxy Version 0.38.1 (Bolger *et al.* 2014) to trim ends and remove short (<100 bp) and poor-quality reads with the preservation of read pair association. A base quality call accuracy of 99.9 % or Phred quality score of 30 was used as the quality cut-off mark. The trimmed reads were then re-subjected to FasqQC analyses to verify that good-quality reads were obtained after trimming. The high-quality trimmed reads were then mapped onto their respective reference genomes using the hierarchical indexing tool, HISAT2, Version 2.1.0 (Kim *et al.* 2015) **[see****Supporting Information—Table S2****]**. The reads from the tomato samples were mapped against the *Solanum lycopersicum* cv. Heinz 1706 (GCA_000188115.3) and those from the sweet pepper samples were mapped to the *Capsicum annuum* cv. Zunla-1 reference genome (GCA_000710875.1).

The BAM files generated from HISAT2 mapping were analysed to determine the mapping quality of reads to the reference genomes using the QualiMap BamQC tool, Version 2.2.2 (Okonechnikov *et al.* 2016). The Phred quality scores of the mapped reads in the BAM files were also determined using the Read Quality tool embedded in the RSeQC package, Version 2.6.4 (Wang *et al.* 2012). Following mapping, featureCounts, Version 2.0.1 was utilized to acquire the raw counts and annotations (Liao *et al.* 2014). Gff annotation files were obtained from NCBI (Agarwala *et al.* 2018). The annotated count data were then subjected to differential gene expression analysis employing the edgeR package, Version 3.24.1, with the trimmed mean of mean values normalization factor (Robinson *et al.* 2009; Liu *et al.* 2015). Differentially expressed genes (DEGs) were classified as genes with false discovery rates (FDRs) and corrected *P*-values of less than 0.05. Downregulated and upregulated genes were labelled as those with log_2_ fold changes of ≤−2 or ≥+2, respectively. The TBtools, Version 1.0971 (Chen *et al.* 2020) software was utilized to construct volcano plots, Venn diagrams and heatmaps.

### *De novo* transcript assembly and differential expression of unmapped reads

The unaligned forward and reverse reads from each sample generated from HISAT2 were used in the *De-Novo* analysis **[see****Supporting Information—Fig. S2****]**. Trinity (Galaxy Version 2.9.1) was used to assemble the unaligned reads employing the inchworm, chrysalis and butterfly packages (Grabherr *et al.* 2011). Initially, all unaligned forward reads were concatenated as well as unaligned reverse reads using the concatenate data set tool (Galaxy Version 1.0.0). The Trinity assembly was assessed for completeness and accuracy using BUSCO (Galaxy Version 5.0.0) and RNA Quast (Galaxy Version 2.2.0). Bowtie2 was subsequently used to map the unaligned paired reads to the Trinity assembly followed by gene counting using the RSEM package (RNA-Seq by Expectation–Maximization) (Galaxy Version 1.1.17) (Grabherr *et al.* 2011). The package TransDecoder (Haas *et al.* 2013) was then implemented to find potential coding regions within the Trinity assembly (Galaxy Version 5.5.0). The .pep file from TransDecoder was used to obtain protein annotations using NCBI BLAST+ blastp (Camacho *et al.* 2009; Cock *et al.* 2015). The tomato .pep and sweet pepper .pep files were analysed using Blastp against the *S. lycopersicum* (https://www.uniprot.org/proteomes/UP000004994) and *C. annuum* (https://www.uniprot.org/proteomes/UP000189700) proteomes embedded in UniProt (Bateman 2019). The hits were filtered to reflect a percent identity and query coverage percentage of ≥80. As previously stated, differential expression was done using the edgeR program.

### Gene ontology and pathway enrichment

Ontological and enrichment analyses categorize important gene clusters engaged in structural, physiological and biological activities. The Blast2Go (Götz *et al.* 2008) tool embedded in the OmicsBox software (OmicsBox 2019) was used for enrichment analysis. Fisher’s exact test was conducted to test the significance of gene ontology (GO) terms whereby GOs with a corrected *P*-value and FDR of ≤0.05 with the Benjamini–Hochberg statistic were considered significantly enriched. Kyoto Encyclopaedia of Genes and Genomes (KEGG) pathway mapper was used to execute pathway enrichment (Kanehisa *et al.* 2019) and KEGG Orthology-Based Annotation System (Xie *et al.* 2011) online tool using the Benjamini–Hochberg FDR correction technique and the hypergeometric test/Fisher’s exact test. Significantly enriched pathways in the list of DEGs were classified as pathways having an FDR-adjusted *P*-value of ≤0.05.

### Validation of DEGs by real-time polymerase chain reaction (qPCR)

The independent gold-standard qPCR assay was conducted to validate the DEGs obtained from the Illumina RNA-Seq analysis. Based on the results, 14 genes were chosen for validation which included those involved in defence pathways and phytohormone biosynthesis. The list also included genes upregulated or downregulated by both SWE treatments but also those upregulated by one SWE and downregulated by the other. *De novo*-aligned transcripts were also validated by qPCR using the WRKY transcription factor and ferredoxin gene. The IDT-Primer Quest tool was used to create the specific primers **[see****Supporting Information—Tables S3****and****S4****]**. The housekeeping gene, actin, was utilized in the validation experiment. A correlation analysis was conducted and the RNA-Seq log_2_ fold change values were plotted against qPCR log fold change values for each treatment group with representative *R*^2^ values. This assay consisted of three biological replicate plants per treatment.

### Analysis of endogenous plant hormones

To investigate the influence of SWEs on endogenous plant hormone levels, leaf samples from three biological replicates of tomato and sweet pepper plants were collected per treatment and analysed for phytohormone levels (betaines, auxins, cytokinins, gibberellins strigolactones and brassinosteroids) at J.H.G. Analytical Services Limited (Waterford, Ireland).

### Assessment of plant defence enzymes

Tomato and sweet pepper plants were foliar-sprayed with AS and SV extracts. Three biological replicate plants were sampled 72 h after treatment for the analysis of plant defence enzymes including chitinase (CHI), β-1,3-glucanase (GLU), phenylalanine ammonia lyase (PAL), peroxidase (POD) and polyphenol oxidase (PPO). The rate of formation of *N*-acetylglucosamine utilizing chitin (crab shells) as the substrate was used to quantify CHI, which was assessed using 585-nm absorbance (Tonon *et al.* 1998). β-1,3-glucanase was measured using absorbance at 500 nm and the amount of glucose released using laminarin as the substrate (Tonon *et al.* 1998). Phenylalanine ammonia lyase activity was determined using absorbance shifts at 290 nm caused by the conversion of *L*-phenylalanine to *trans*-cinnamic acid (Sila *et al.* 2016). Peroxidase activity was measured at 420-nm absorbance using the substrate pyrogallol (Hammerschmidt *et al.* 1982). Polyphenol oxidase activity was analysed by measuring changes in absorbance at 495 nm using the substrate catechol (Ali *et al.* 2021b).

### Effects of SWEs on disease reduction and plant growth

The trial consisted of 60 plants per treatment and was outlined as previously mentioned in the greenhouse section above. Plants were foliar-treated with 0.5 % SWE as previously mentioned. To confirm the elicitation effects, 6 h after treatment, 30 plants per treatment were foliar-spray-inoculated with a bacterial cell suspension of *Xanthomonas campestris* pv. *vesicatoria* (1.5 × 10^8^ CFU per mL) or a conidial suspension of *Alternaria solani* (1 × 10^6^ spores per mL) (Ali *et al.* 2021b). *Xanthomonas campestris* pv. *vesicatoria* and *A. solani* are the causal agents of bacterial leaf spot and early blight, respectively. The plants were subsequently kept under humid conditions (48 h) in the greenhouse for the infection to set. Additional SWE treatments were applied every 10 days until the end of the crop stage. The percent of disease was scored using a disease rating scale by Ali *et al.* (2016) (1 = 0 %; 2 = 1–10 %; 3 = 11–25 %; 4 = 26–40 %; 5 = 41–55 %; 6 ≥ 56 %). The other uninfected plants (30 plants per treatment) were treated with the SWEs as previously mentioned. At the active bearing stage, chlorophyll content of mature leaves at the fourth node was assessed utilizing a chlorophyll meter (atLEAF+, FT Green LLC, USA) along with plant height, fruit yield and total dry biomass.

### Analysis of plant nutrients

Leaves and roots from three biological replicates of tomato and sweet pepper plants were collected from each treatment and sent to Agro Services International, FL, USA, for the analysis of plant nutrient levels. The nutrient elements quantified included calcium, nitrogen, potassium, sodium, magnesium, copper, sulphur, boron, copper, phosphorus, manganese and zinc.

### Statistical analysis

All data sets excluding the sequencing data were analysed using IBM SPSS Statistical software package, Version 27 (IBM Corporation 2011). The significance amongst the groups was tested by one-way analysis of variance (ANOVA) followed by LSD *post hoc* analysis (*P* < 0.05). Pearson correlation analysis was carried out to test the linear association between chosen RNA-Seq DEGs and the qPCR results. A probability value of less than 0.05 was considered a significant difference.

## Results

### Compositional analysis of *A. spicifera* and *S. vulgare* extracts

Table 1 gives an insight into various bioactive compounds present in both extracts. The chemical components included mannitol, alginic acid, laminarins and fucoidan as well as total polysaccharides and carbohydrates. It also gives quantitative measures of protein and fat content, fibre, oils and other important nutrients. From Table 1, the bioactive compounds with the highest concentration observed were alginic acid in both AS (11.6 %W) and SV (9.4 %W) extracts followed by fucoidans.

**Table 1.** Bioactive compositional profiling of *A. spicifera* and *S. vulgare* extracts.

Parameter	Type of analysis	Reported levels in SWEs	
		*A. spicifera* (%W)	*S. vulgare* (%W)
Mannitol	HPLC–PDA	4.800	5.102
Alginic acid	HPLC–PDA	11.558	9.375
Laminarins	HPLC–PDA	5.025	4.460
Fucoidan	GC–MS	6.124	5.245
Protein content	Kjeldahl digestion	12.818	9.990
Fat content	Soxhlet extraction	2.345	2.160
Saturates	HPLC–PDA	0.556	0.560
Unsaturates	HPLC–PDA	1.724	1.600
Omega-3 oils	HPLC–PDA	0.400	0.450
Omega-6 oils	HPLC–PDA	1.300	1.150
Carbohydrates	HPLC–PDA	30.500	28.800
Polysaccharides	HPLC–PDA	27.500	24.200
Dietary fibre content	Digestion/gravimetry	38.900	35.500
Iodine as I^2^	Ion chromatography	71.000	112.00

The key components include total polysaccharides and carbohydrates as well as total fucoidan, laminarin, mannitol and alginic acid content.

Table 2 illustrates hormone profiling in the SWEs. Cytokinin content dominated the other hormones tested in both extracts, of which *A. spicifera* had 45.00 mg kg^−1^ and *S. vulgare* had 29.00 mg kg^−1^. Gibberellins and auxins were also present in high quantities compared to the other hormones tested. Betaines and strigolactones were much lower and brassinosteroids accounted for the lowest hormone level of less than 0.002 mg kg^−1^ in both extracts.

**Table 2.** Hormonal content of *A. spicifera* and *S. vulgare* SWEs.

Parameter	Type of analysis	Reported levels in SWEs	
		*A. spicifera* (mg kg^−1^)	*S. vulgare* (mg kg^−1^)
Betaines	HPLC–PDA	0.023	0.014
Auxins	HPLC–PDA	23.000	13.000
Cytokinins	HPLC–PDA	45.000	29.000
Gibberellins	GC–MS	33.000	17.000
Strigolactones	Kjeldahl digestion	0.246	0.085
Brassinosteroids	Soxhlet extraction	<0.002	<0.002

### Overview of RNA-Seq mapping statistics and general DEG profiling for tomato and sweet pepper

**Figure 1.**
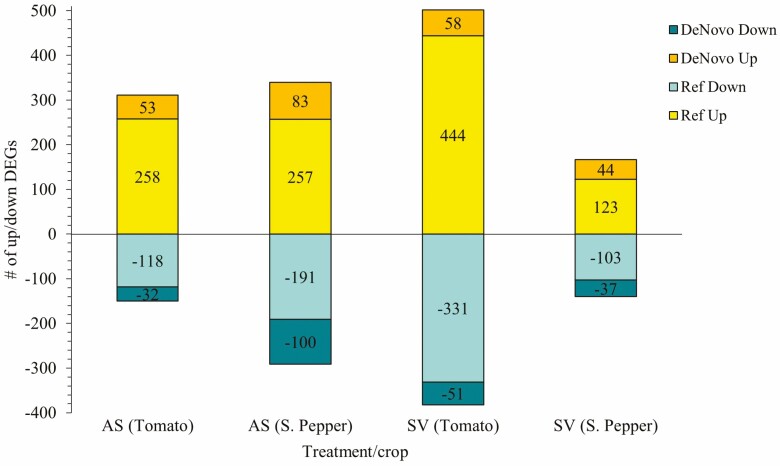
Total up- and downregulated gene profiles for tomato and sweet pepper plants due to treatment with SWEs. The DEG profiles are based on both reference-based and *de novo*-based analyses. The criteria used for significantly expressed DEG filtering were *α* ≤ 0.05, FDR ≤ 0.05 and fold change ≥+2 for upregulated and ≤−2 for downregulated genes.

**Figure 2.**
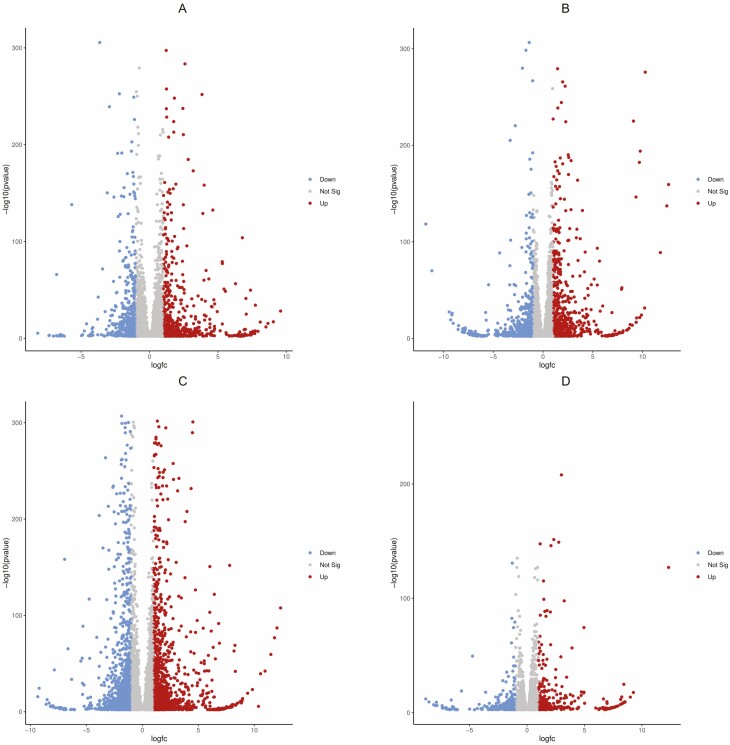
Volcano plot highlighting changes in the regulation of genes in SWE-treated plants. Genes that were upregulated (red), downregulated (blue) or had no significant changes (grey) due to the application of SWE. The graph plotted denotes the *P*-value on the vertical axis on a logged 10 scale against gene expression ratios (log_2_ fold change) on the horizontal axis on a logged 2 scale. The criteria used for DEG filtering were *α* ≤ 0.05, FDR ≤ 0.05 and fold change ≥+2 for upregulated and ≤−2 for downregulated. A = AS tomato; B = AS S. pepper; C = SV tomato; D = SV S. pepper.

Sequencing read and mapping statistics are highlighted in **Supporting Information—Tables S1****and****S2**. It can be noted that over 98 % of all reads for all samples were retained after quality filtering of Phred score of 30 and above **[see****Supporting Information—Fig. S3****]**. There was a low error rate for all samples (0.03 %) and the mapping rate was generally higher for tomato > 90 % compared to sweet pepper > 85 %. The remaining unaligned reads were assembled and analysed using the Trinity *de novo* algorithm. Figure 1 illustrates the general upregulated and downregulated transcript profiles of treatment groups for both reference- and *de novo*-based analysis. A log_2_ fold change of ≥+2 for upregulated and ≤−2 for downregulated, and a *P*-value and FDR of ≤0.05 were used for filtering DEGs. Tomato plants treated with SV had the overall highest DEGs compared to the other treatment groups. In comparison to the other groups, SV-treated sweet pepper plants exhibited the lowest DEG profile. Furthermore, there were more upregulated genes in all four groups for both reference-based and *de novo*-based analysis compared to downregulated DEGs. The red SWE (AS) treatments had similar proportions in DEGs in both crops compared to SV-treated plants (Fig. 2). Most reads aligned to the *S. lycopersicum* and *C. annuum* references **[see****Supporting Information—Fig. S4****]** with most enzyme classes being associated with oxidoreductases, transferases and hydrolases **[see****Supporting Information—Fig. S5****]** in the list of transcripts. All downstream analyses were carried out with combined *de novo*- and reference-based results.

### Clustering and heatmap visualization of DEG profiles

**Figure 3.**
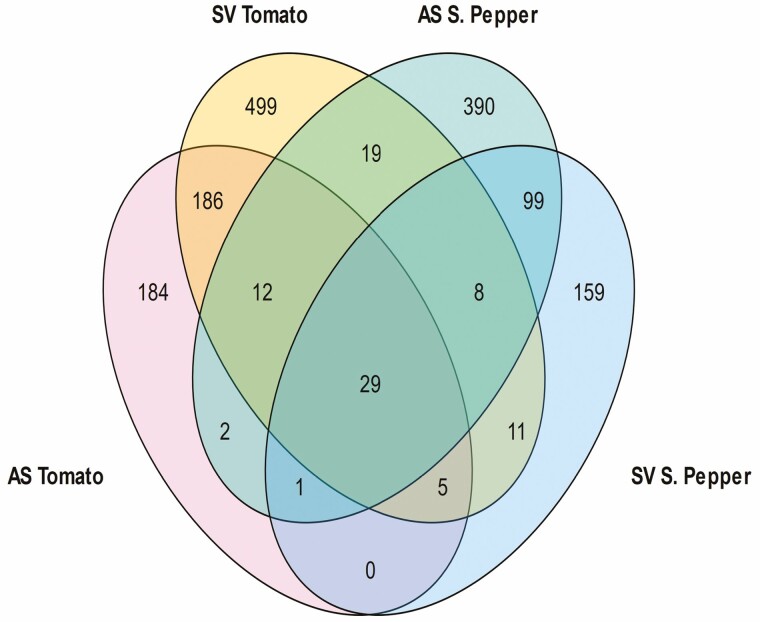
Venn diagram highlighting gene regulation profiles of commonly expressed genes in SWE-treated plants. The list represents only common genes annotated in both crops.

**Figure 4.**
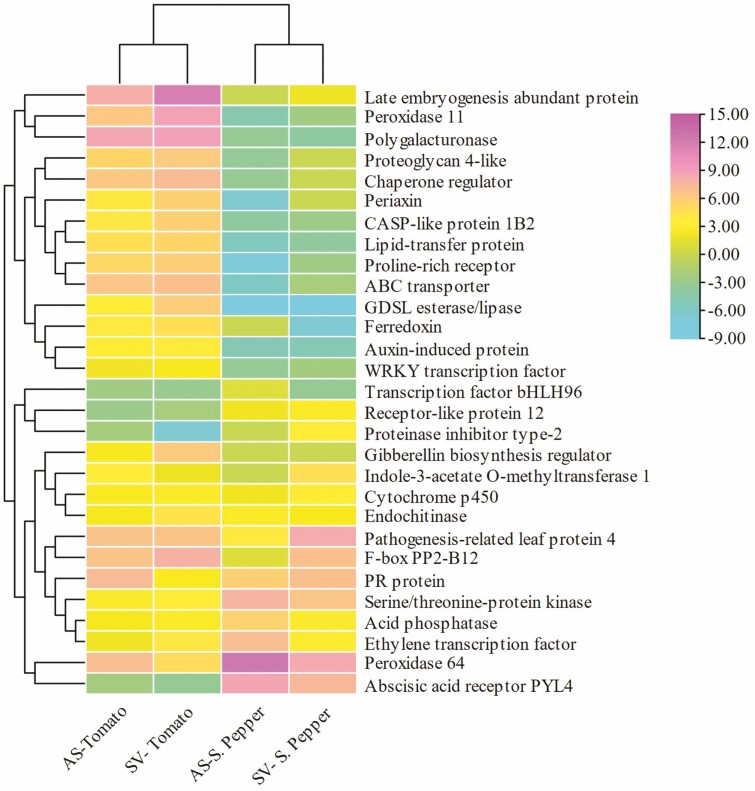
Heatmap of expressed genes of upregulated or downregulated in all four treatment groups. Rows and columns were clustered using correlation distance and average linkage.

The Venn diagram shown in Fig. 3 illustrates 29 common annotated transcripts for all four treatment groups. There were 499, 159, 184 and 390 unique DEGs occurring in SV tomato, SV sweet pepper, AS tomato and AS sweet pepper, respectively. A heatmap shows the 29 DEGs in the four treatment groups, for tomato and sweet pepper where two distinct clusters were observed. A clear pattern was shown for tomato and sweet pepper, where the upregulation of one set of DEGs in one crop was accompanied by the downregulation of the same DEGs in the other crop (Fig. 4). Peroxidase 64 and the late embryogenesis abundant protein genes were the highest upregulated DEGs in the list, whereas GDSL esterase/lipase and the receptor-like protein 12 were the most downregulated transcripts. However, GDSL esterase/lipase was highly downregulated in sweet pepper but upregulated in tomato. Furthermore, the receptor-like protein 12 was highly downregulated in tomato but upregulated in sweet pepper.

### GO and KEGG pathway enrichment analysis for DEG profiles in tomato and sweet pepper

**Figure 5.**
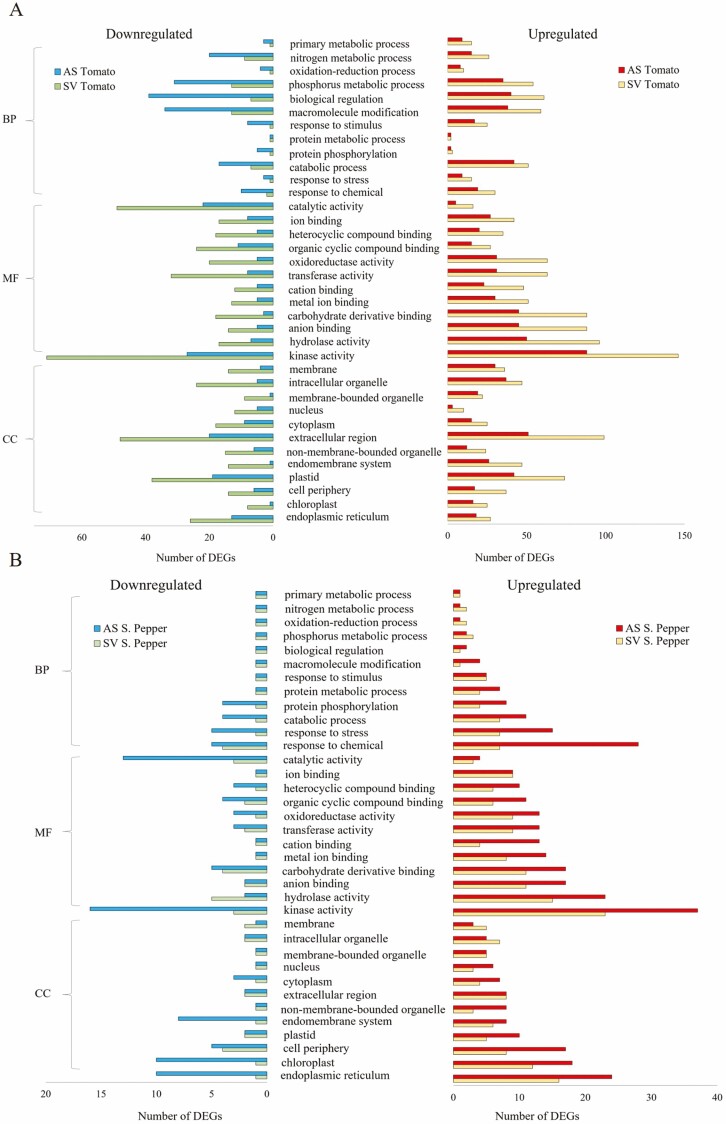
Number of significantly enriched GO terms for biological processes (BP), molecular functions (MF) and cellular components in tomato (A) and sweet pepper (B).

**Figure 6.**
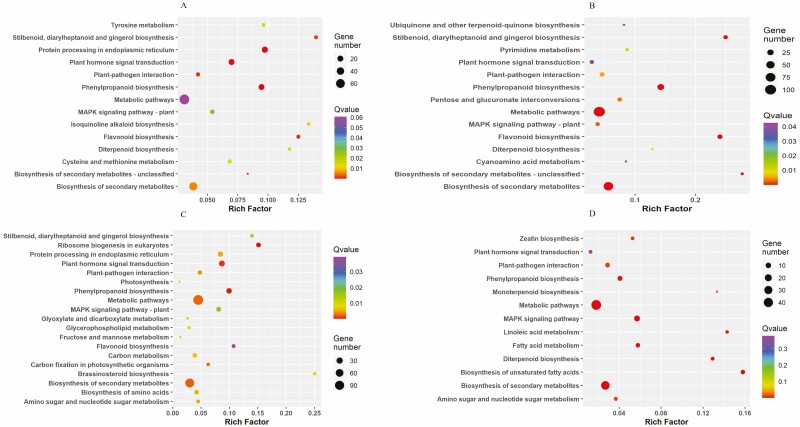

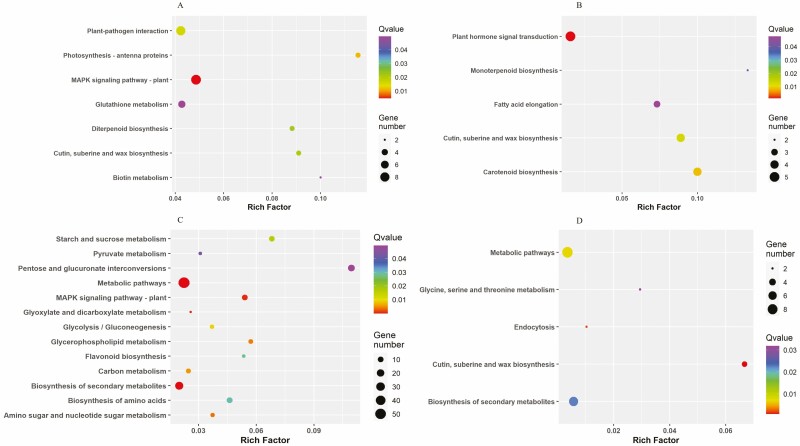
(i) Rich factor bubble plot of significantly enriched KEGG pathway statistics for upregulated genes. The rich factor represents the ratio of the DEG number to the total genes in a certain pathway. The *Q*-value represents the corrected *P*-value ranging from 0 to 1. The colour and size of the bubbles represent the range of the *Q*-value and the number of DEGs mapped to the indicated pathways, respectively. The top enriched pathways are shown for the upregulated gene list. A = AS tomato; B = AS S. pepper; C = SV tomato; D = SV S. pepper. (ii) Rich factor bubble plot of significantly enriched KEGG pathway statistics for downregulated genes. The rich factor represents the ratio of the DEG number to the total genes in a certain pathway. The *Q*-value represents the corrected *P*-value ranging from 0 to 1. The colour and size of the bubbles represent the range of the *Q*-value and the number of DEGs mapped to the indicated pathways, respectively. The top enriched pathways are shown for the downregulated gene list. A = AS tomato; B = AS S. pepper; C = SV tomato; D = SV S. pepper.

Figure 5A and B illustrates the various enriched GO terms in the three main ontological groupings (biological processes, BP; molecular functions, MF; and cellular components, CC) for tomato and sweet pepper. The Fisher’s exact test was used to define the significant enhancement of GO terms, with GOs having a corrected *P*-value and FDR of ≤0.05 based on the Benjamini–Hochberg method (Haynes 2013). As seen in Fig. 5A for tomato, most enriched terms belonged to BP including biological regulation, macromolecule modification and phosphorous metabolism. Some of the highly enriched MF terms included catalytic activity, kinase and hydrolase activity. The main enriched terms for CC included the extracellular region and cytoplasm. Contrastingly, in sweet pepper (Fig. 5B), responses to chemicals, stress, catabolic processes and protein phosphorylation were the top BP in both upregulated and downregulated DEGs. The most substantially enriched GO keywords for MF in sweet pepper were kinase activity, carbohydrate derivative binding and organic cyclic compound binding. For CC, the endoplasmic reticulum, chloroplast and endomembrane systems were the most enriched terms for both upregulation and downregulation in sweet pepper. According to the hypergeometric test/Fisher’s exact test and the FDR Benjamini–Hochberg correction technique, a rich factor bubble plot was created to illustrate significant KEGG pathway statistics. Significantly enriched pathways were classified as those with an FDR-adjusted *P*-value of ≤0.05. In Fig. 6i, it can be seen that SV tomato had the greatest number of enriched terms, as expected since SV tomato had the greatest number of DEGs. The majority of terms belonged to metabolic pathways and the biosynthesis of secondary metabolites. The more important significantly enriched terms included DEGs engaged in phenylpropanoid biosynthesis, plant hormone signal transduction, plant–pathogen interaction, MAPK signalling and photosynthesis to name a few (Fig. 6i). The downregulated KEGG terms seen in Fig. 6ii were much lower in numbers when compared to upregulated terms in Fig. 6i. Most DEGs for the downregulated list fell within the same metabolic pathways and under biosynthesis of secondary metabolites.

### Correlation analysis of DEGs quantified by RNA-Seq and the qPCR assay

**Figure 7.**
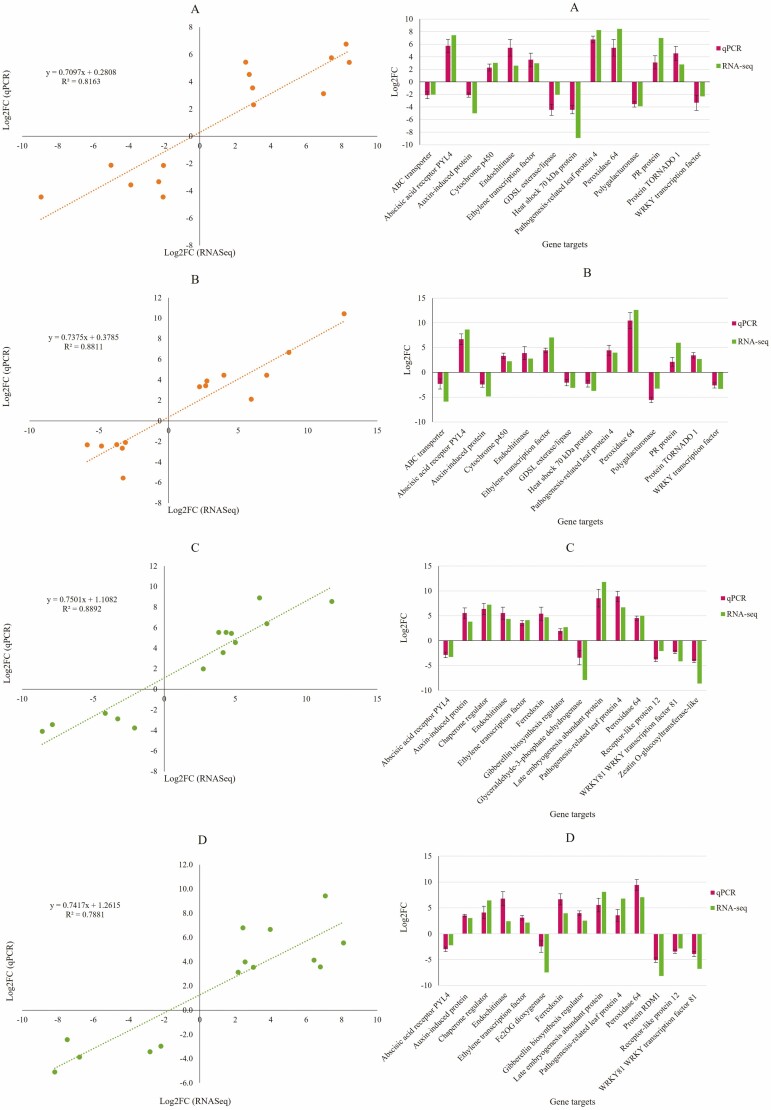
qPCR validation of DEGs from the RNA-Seq analysis. A = SV S. pepper; B = AS S. pepper; C = SV tomato; D = SV tomato. RNA-Seq log_2_ fold change was plotted against qPCR log fold change values and correlation represented by the *R*^2^ value on each plot. Actin was used as the reference housekeeping gene. The bar graphs highlight the selected candidate genes with their represented log_2_FC values with vertical bars representing SD (mean = 3 biological replicates).

Figure 7 shows a positive correlation between the results of the DEG quantification by RNA-sequencing experiment and the qPCR of gene expression assay. The actin gene was chosen as the reference housekeeping gene for the qPCR experiment. Primer efficiency ranged between 98 and 99 %. All plots in Fig. 7 had *R*^2^ values of 0.75 or greater, signifying a strong positive correlation. Figure 7C (SV tomato) had the strongest correlation (88.9 %) and Fig. 7D (AS tomato) had the lowest (78.8 %).

### Effect of SWEs on endogenous phytohormone levels in plants

**Figure 8.**
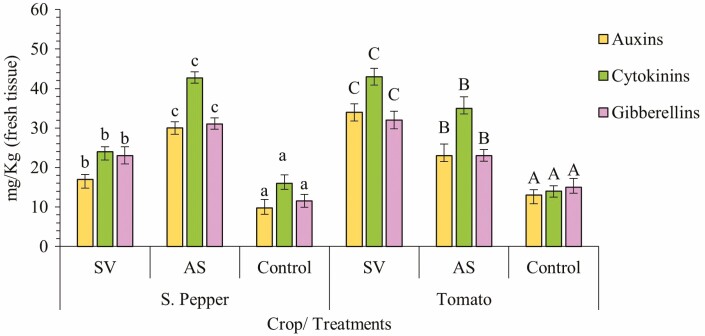
Plant hormone levels in sweet pepper and tomato leaves after treatment with SWEs. The data represent the mean level of auxins, cytokinins and gibberellins from three replicate plants per treatment. The data represent the mean of three plants and vertical bars represent standard deviation. The significance was tested by one-way ANOVA followed by LSD *post hoc* analysis (*P* < 0.05). Different letters denote a significant difference between groups where *P* < 0.05. The analysis also tested for betaines, strigolactones and brassinosteroids but quantities were not significantly different among treatments and were much lower compared to the other three hormones **[see****Supporting Information—Table S5****]**. Statistical tests were done on crops separately.

In an effort to study the implication of SWE foliar sprays on endogenous hormonal levels of plants, a hormonal profiling assay was done simultaneously with the RNA sequencing. This was carried out to compare and relate DEGs to metabolite changes in the plant. Figure 8 shows that the SWE treatments (AS and SV) in tomato and sweet pepper led to an enhanced accumulation of three important phytohormones including auxins, cytokinins and gibberellins as compared to control plants. Cytokinin content was the highest, followed by gibberellins and auxins. All three hormone levels were significantly higher than the control according to ANOVA and LSD analysis (*P* ≤ 0.05). However, the levels of betaines, strigolactones and brassinosteroids were not significantly different in comparison to the control group **[see****Supporting Information—Table S5****]**. Brassinosteroids were at the lowest levels in relation to the other five hormones for both crops (<0.002 mg kg^−1^).

### Effect of SWE on defence enzyme activities

**Figure 9.**
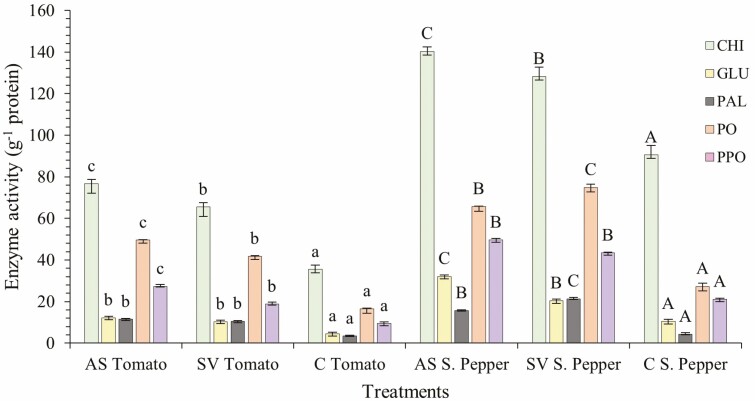
Effects of AS and SV SWEs on tomato and sweet pepper defence enzyme activities 72 h after foliar treatment. The data represent the mean of three plants and vertical bars represent standard deviation. The significance was tested by one-way ANOVA followed by LSD *post hoc* analysis (*P* < 0.05). Different letters denote a significant difference between groups where *P* < 0.05. Statistical tests were done on crops separately.

The activities of CHI, GLU, PAL, POD and PPO were all significantly higher (*P* < 0.05) in SWE-treated tomato and sweet pepper plants after 72 h of foliar treatment (Fig. 9). The activity of CHI was the overall highest, whereas activity of PAL was the lowest. Overall sweet pepper plants had higher enzyme activity compared to tomato plants.

### Effects of SWEs on disease reduction and plant growth

Application of both SWEs was able to significantly reduce disease levels (*P* < 0.05) (Table 3) of bacterial spot and early blight while simultaneously leading to significant increases (*P* < 0.05) in plant height, chlorophyll content and fruit harvest (Table 4) in both tomato and sweet pepper crops.

**Table 3.** Effect of application of SWEs on disease severity of bacterial leaf spot and early blight of tomato and sweet pepper plants.

Treatments	Bacterial leaf spot		Early blight	
	Tomato	S. pepper	Tomato	S. pepper
Control	67.16 ± 1.83a	60.01 ± 1.71a	58.39 ± 1.56a	50.97 ± 1.69a
SV	40.13 ± 1.32b	47.84 ± 1.36b	38.36 ± 1.69b	31.05 ± 1.20b
AS	32.11 ± 2.06c	37.99 ± 1.61c	27.47 ± 1.98c	29.93 ± 1.79c

The data represent the mean ± SD of *n* = 30 plants. Significance was tested by one-way ANOVA followed by LSD *post hoc* analysis (*P* < 0.05). Different letters denote a significant difference between groups where *P* < 0.05. Statistical tests were done on each crop separately.

**Table 4.** Effect of foliar application of SWEs on plant growth parameters (plant height, chlorophyll content, total fruit yield).

Treatments	Plant height (cm)		Chlorophyll content		Total fruit yield (kg)	
	Tomato	S. pepper	Tomato	S. Pepper	Tomato	S. Pepper
Control	83.72 ± 1.65a	66.77 ± 2.21a	42.95 ± 1.24a	45.19 ± 1.74a	17.26 ± 1.24a	18.00 ± 1.22a
SV	99.05 ± 1.96b	80.16 ± 1.78b	50.93 ± 1.34b	55.25 ± 1.53b	22.72 ± 1.38b	27.55 ± 1.52b
AS	110.97 ± 1.55c	86.36 ± 1.73c	51.83 ± 1.60c	57.27 ± 1.57c	23.56 ± 1.02b	30.16 ± 1.83c

The data represent the mean ± SD of *n* = 30 plants. Total fruit yield harvest was from 30 plants. Significance was tested by one-way ANOVA followed by LSD *post hoc* analysis (*P* < 0.05). Different letters denote a significant difference between groups where *P* < 0.05. Statistical tests were done on each crop separately.

### Effects of SWEs on nutrient and mineral content of tomato and sweet pepper roots and shoots

Analyses were done to test the effects of AS and SV extracts on plant nutrient profiles. Generally, both SWEs led to increased nutrient profiles of both tomato and sweet pepper. Furthermore, both root and shoot tissue showed a substantial rise in nutritional content.

Calcium, sulphur, boron, copper, iron, manganese and zinc were all significantly enriched in tomato shoots compared to control plants (Table 5). Interestingly, nitrogen was only significantly enhanced in tomato shoots with AS treatment, whereas magnesium and phosphorous were only increased in SV-treated tomato shoots. Both sodium and potassium remained unchanged in tomato shoots (Table 5).

**Table 5.** Analysis of nutrient profile of shoots of tomato and sweet pepper treated with AS and SV extracts.

	Tomato			S. pepper		
Nutrient element	AS	SV	Control	AS	SV	Control
Calcium (%)	3.80 ± 0.030c	2.50 ± 0.010b	1.65 ± 0.050a	4.10 ± 0.901c	3.33 ± 0.901b	1.33 ± 0.113a
Magnesium (%)	0.53 ± 0.001a	0.63 ± 0.001b	0.67 ± 0.004b	0.37 ± 0.002ns	0.34 ± 0.021ns	0.35 ± 0.004ns
Potassium (%)	2.19 ± 0.010ns	2.22 ± 0.020ns	2.20 ± 0.030ns	3.22 ± 0.991c	2.76 ± 0.121b	2.11 ± 0.098a
Sodium (%)	0.07 ± 0.001ns	0.05 ± 0.001ns	0.05 ± 0.005ns	0.04 ± 0.002ns	0.03 ± 0.002ns	0.03 ± 0.004ns
Nitrogen (%)	6.22 ± 0.070b	4.59 ± 0.072a	4.55 ± 0.065a	5.66 ± 1.021c	5.12 ± 0.111b	3.55 ± 0.005a
Phosphorus (%)	0.25 ± 0.004a	0.39 ± 0.002b	0.21 ± 0.001a	0.12 ± 0.002a	0.43 ± 0.014b	0.11 ± 0.132a
Sulphur (%)	0.09 ± 0.003b	0.09 ± 0.004b	0.04 ± 0.004a	0.04 ± 0.001ns	0.03 ± 0.001ns	0.02 ± 0.001ns
Boron (ppm)	38.0 ± 0.340c	32.0 ± 0.440b	27.0 ± 0.670a	26.12 ± 1.102b	23.34 ± 0.032a	22.12 ± 0.322a
Copper (ppm)	81.9 ± 1.230c	78.9 ± 0.450b	62.7 ± 0.550a	99.12 ± 1.112c	76.22 ± 1.021b	55.43 ± 1.221a
Iron (ppm)	810 ± 1.003c	703 ± 1.012b	237 ± 1.050a	777.34 ± 1.212c	712.22 ± 1.421b	201.21 ± 1.092a
Manganese (ppm)	88 ± 0.201c	36 ± 0.111b	30 ± 0.431a	76.45 ± 1.032c	55.43 ± 1.121b	23.23 ± 1.112a
Zinc (ppm)	57 ± 0.053c	51 ± 1.021b	31 ± 1.112a	45.32 ± 1.218c	40.21 ± 1.081b	22.23 ± 0.443a

The data represent the mean ± SD of *n* = 3 plants. Significance was tested by one-way ANOVA followed by LSD *post hoc* analysis (*P* < 0.05). Different letters denote a significant difference between groups where *P* < 0.05 and ns denotes no significant difference. Statistical tests were done on crops separately.

In sweet pepper-treated shoots, calcium, potassium, nitrogen, copper, iron, manganese and zinc levels significantly increased (Table 5). Only boron level was significantly greater in AS-treated pepper and tomato shoots compared to the control. The level of phosphorous was significantly higher in SV-treated sweet pepper shoots than in the control. Both tomato and sweet pepper plant shoots treated with SV extract showed this change in phosphorus levels.

In tomato roots, the levels of calcium, magnesium, potassium, nitrogen, phosphorous, sulphur, boron, copper, iron, manganese and zinc significantly increased in SWE-treated tomato plants. Boron, however, was significantly increased only in SV-treated tomato roots (Table 6). Further, calcium, magnesium, potassium, nitrogen, phosphorous, sulphur, copper, iron, manganese and zinc levels significantly increased in SWE-treated sweet pepper roots, whereas sodium and boron remained unchanged (Table 6).

**Table 6.** Analysis of root nutrient profile in tomato and sweet pepper treated with AS and SV extracts.

	Tomato			S. pepper		
Nutrient element	AS	SV	Control	AS	SV	Control
Calcium (%)	2.72 ± 0.111b	2.58 ± 0.012b	1.99 ± 0.042a	3.11 ± 0.121b	3.10 ± 0.111b	1.43 ± 0.439a
Magnesium (%)	0.71 ± 0.002c	0.65 ± 0.004b	0.31 ± 0.022a	0.70 ± 0.002b	0.77 ± 0.021b	0.44 ± 0.053a
Potassium (%)	2.90 ± 0.102c	2.21 ± 0.121b	1.13 ± 0.212a	4.45 ± 0.195b	4.11 ± 0.227b	3.01 ± 0.045a
Sodium (%)	0.06 ± 0.003ns	0.05 ± 0.002ns	0.04 ± 0.001ns	0.03 ± 0.001ns	0.03 ± 0.004ns	0.04 ± 0.002ns
Nitrogen (%)	4.79 ± 0.111c	4.37 ± 0.212b	4.23 ± 0.320a	5.01 ± 0.231b	5.11 ± 0.022b	3.98 ± 0.981a
Phosphorus (%)	0.30 ± 0.002b	0.34 ± 0.002b	0.21 ± 0.003a	0.41 ± 0.002b	0.44 ± 0.004b	0.31 ± 0.025a
Sulphur (%)	0.14 ± 0.003c	0.08 ± 0.001b	0.01 ± 0.020a	0.11 ± 0.120b	0.09 ± 0.004b	0.02 ± 0.042a
Boron (ppm)	26 ± 0.221a	34 ± 0.902b	25 ± 0.859a	21.21 ± 0.550ns	25.22 ± 0.333ns	23.22 ± 0.227ns
Copper (ppm)	56.6 ± 0.298b	54.8 ± 0.221b	50.1 ± 0.776a	61.21 ± 0.216c	56.33 ± 0.236b	44.33 ± 0.389a
Iron (ppm)	1179 ± 1.112c	1157 ± 1.021b	1000 ± 1.002a	1323 ± 0.878c	1190 ± 0.228b	1050 ± 0.128a
Manganese (ppm)	114 ± 0.322b	113 ± 1.121b	76 ± 1.111a	210 ± 0.341b	209 ± 0.023b	188 ± 0.820a
Zinc (ppm)	83 ± 1.001b	70 ± 1.043b	55 ± 0.667a	99.11 ± 0.002b	93.22 ± 0.221b	76.34 ± 0.238a

The data represent the mean ± SD of *n* = 3 plants. Significance was tested by one-way ANOVA followed by LSD *post hoc* analysis (*P* < 0.05). Different letters denote a significant difference between groups where *P* < 0.05 and ns denotes no significant difference. Statistical tests were done on crops separately.

In general, AS-treated plants had significantly higher levels of calcium in leaves than SV and control plants. Higher levels of calcium were also seen in roots of AS-treated plants, but were not significantly different from SV-treated plants. Generally, the levels of calcium, potassium and nitrogen were higher than the other macronutrients. In general, the iron content remained the highest among the micronutrients in the SWE-treated plants. For example, there was a 241.77 % increase in AS-treated tomato leaves and a 196.62 % increase in SV-treated tomato leaves for iron content (Table 5).

## Discussion

### Overview of the transcriptomic response in plants due to SWE priming

The transcriptome snapshot after 72 h of foliar treatment with two different SWEs was evaluated for tomato and sweet pepper plants. The RNA sequencing and bioinformatic analysis showed significant enrichment of genes involved in processes that affect plant growth and defence capability including photosynthesis, carbon and nitrogen metabolism, plant hormone signal transduction, plant–pathogen interaction, secondary metabolite metabolism, MAPK signalling, amino acid biosynthesis and several other metabolic networks. The upregulation of these genes can be therefore linked to greater plant biomass, yield and stress resistance in SWE-treated plants. Based on gene homology annotation, there were 29 significantly expressed genes (DEGs) in the four treatment groups, and of these, peroxidase 64 was the highest upregulated gene. The accumulation of peroxidases has been linked to increased auxin metabolism, formation of suberin and lignin, cell wall cross-linking, synthesis of phytoalexins and reactive oxygen species (ROS) and reactive nitrogen species metabolism (Almagro *et al.* 2009). Reactive oxygen species have been identified as important molecules in the priming defence mechanism of plants (Borges *et al.* 2014). Interestingly, the GDSL esterase/lipase gene was one of the most downregulated overall, but only in sweet pepper. Contrastingly this gene was upregulated in tomato. The GDSL esterase/lipase gene has been linked to multifunctional processes including plant growth and defence, and also resistance to the necrotrophic fungus *Alternaria brassicicola*, mainly through Et-mediated signalling mechanisms (Chepyshko *et al.* 2012). The receptor-like protein 12 was highly downregulated in tomato but was upregulated in sweet pepper. This receptor gene has also been associated with various signalling transduction mechanisms which led to growth and increased defence responses (Lv *et al.* 2016). Besides these two genes, several other genes followed similar patterns of upregulation in one plant species and downregulation in the other species, as shown in the heatmap. This is usually the case with complex plant systems as they exhibit varied responses to external stimuli even though they may belong to the same family.

### Plant defence responses

Many unique bioactive chemicals found in SWEs, such as ulvans, laminarins and carrageenans, have been demonstrated to trigger plant defence mechanisms against a wide range of diseases (Cluzet *et al.* 2004; Bajpai *et al.* 2019). These bioactive molecules can serve as priming agents or pathogen-associated molecular patterns (PAMPs), which therein leads to Induced Systemic Resistance (ISR) and Systemic Acquired Resistance (SAR) responses. The results showed that SWE-primed plants had higher amounts of pathogenesis-related protein synthesis genes (e.g. *PR*, *PR4*, *PR4-A*, *PR-5*, *PR-STH-2*) which therefore points towards SA signalling for defence. Many other genes were also upregulated that are known to contribute towards pathogenic defence and even alleviation of abiotic and biotic stress. The pathogenesis-related gene transcriptional activator *PTI5* was highly upregulated in both crop plants by both SWE treatments. This gene has been documented to play roles in *Pto*-mediated resistance and was directly linked to *Pseudomonas syringae* resistance in tomato (He *et al.* 2001). The genes associated with the late embryogenesis abundant protein was the second highest upregulated gene in the experiment. This gene has been linked to many protective functions in plants, including aiding in drought, salinity and cold stress (Chen *et al.* 2019).

Additionally, auxin biosynthesis genes were also reported as highly upregulated, and even though most studies show this phytohormone to be a plant growth regulator, it also plays a role in plant defence (Fu and Wang 2011). Furthermore, the abscisic acid receptor *PYL4*-like was also identified in the experiment as highly upregulated by both extracts. This gene has been linked with JA signalling and has been shown to confer downstream resistance by signal transduction facilitated by ABA-activated protein kinases (García-Andrade *et al.* 2020).

The initiation of some of these key genes and other phytohormone-related genes implies that both the AS and SV extracts led to a prime-triggered plant signalling cascade of several phytohormone signalling pathways. Additionally, receptor-like kinases, for example, LRR receptor-like serine/threonine-protein kinases, were also upregulated across both plant models with AS and SV treatments. These kinases are known to play an important role in defensive response signalling as well as developmental control mechanisms and regulation of a myriad of cellular activities (Afzal *et al.* 2008; Lee *et al.* 2017).

### Cell wall modification

Interestingly, in the current study, there were cell wall biosynthesis-related transcripts that were highly upregulated after SWE application in both crops. For example, there were several MYB transcription factor genes highly upregulated and these have been directly linked to secondary cell wall formation together with cellulose and lignin synthesis (Miedes *et al.* 2014). Another example was the upregulation of class III peroxidases which have been associated with secondary cell wall biosynthesis (Lin *et al.* 2016). All these mechanisms would favourably contribute to the plant’s defence capacity.

### Trade-off between defence and growth of plants

In an effort to better implement agricultural practices, one must acknowledge the implications for increased plant growth while simultaneously maintaining a defensive fort against attacking pathogens. There was a balanced trade-off observed in the current study between plant development and the prevalence of different defensive mechanisms. This was evident from the transcriptional profile which revealed the upregulation of genes such as *ETR1*, *PR1a*, *PinII*, etc., whilst plant growth continued to increase significantly compared to control plants. Priming or re-treating plants have been shown to be effective regimes for reaching essential growth and stable defence mechanism goals (Bajpai *et al.* 2019; Islam *et al.* 2020). Priming of plants is an intuitive, adaptive and even low-cost mechanism that aids in the effective stimulus of faster, stronger and more lasting defence inducible effects. This type of balancing strategy is also connected to enhanced biotic and abiotic tolerance in crops (Martinez-Medina *et al.* 2016). With a large number of genes associated with redox signalling and sensing, the transcriptome changes observed in this study confirm the priming effect of the SWEs. Additionally, based on the cellular and molecular GO terms such as transcription factor activities, as well as chaperones and folding catalysts, there was a clear pattern of priming of plants against future infections. The chaperones are involved in proper protein folding which would drive the translation of proteins and help with the precise folding and maintenance leading to the optimal functioning of the proteins. Collectively, the results demonstrated how effective the SWEs can be as priming agents on plants because of their diverse composition of bioactive compounds which are capable of evoking multiple favourable effects in plants.

### Induction of oxidative phosphorylation and photosynthesis-related genes

Photosynthesis is a crucial frontier for plant growth, development and production (Kohli *et al.* 2020). Seaweed extracts were shown to upregulate several genes associated with photosynthetic activities in the current investigation. Seaweed extract treatment significantly upregulated the cytochrome c oxidase subunit 6a. This gene belongs to the mitochondrial electron transport chain in the oxidative phosphorylation pathway. Cytochrome c oxidase is the module of the respiratory chain which catalyses the reduction of oxygen to water and is of utmost importance in providing cellular energy and sets and maintains metabolic homeostasis (Radin *et al.* 2015; Wilson 2017). Ferredoxin was highly upregulated by the SWEs in the current study. Ferredoxin (Fds) are directly involved in nitrogen and sulphur metabolism (Knaff and Hirasawa 1991), as well as enzymes involved in secondary metabolism (Brouquisse *et al.* 1989). Fds also contribute electrons to the reduction of NADP via the Ferredoxin-NADP+-Reductase (Knaff and Hirasawa 1991). Fds are also involved in redox-controlled chloroplastic enzyme modulation through flow towards ferredoxin-thioredoxin-reductase and thioredoxins (Knaff and Hirasawa 1991). Additionally, they play critical roles in electron dissemination and the preservation of the redox environment of the stroma (Holtgrefe *et al.* 2003) as well as in nitrogen and sulphur assimilation, chlorophyll biosynthesis, and the synthesis of phytochrome and fatty acids (Hanke and Mulo 2013).

The current study also showed a vastly upregulated chloroplastic ATP synthase gene (>3 fold change) by the SWEs. This gene is critical as it is responsible for the majority of cellular ATP production and is referred to as a ‘rotary enzyme’ (McCarty *et al.* 2000). A recent study indicated that, besides contributing to ATP synthesis, the upregulation of the alpha-subunit of the ATP chloroplastic synthase gene contributed to strengthening plant resistance towards *Botrytis cinerea* (grey mould) as well as development of broad-spectrum resistance in tobacco (Gong *et al.* 2021).

### Stilbenoid, diarylheptanoid and gingerol biosynthesis

The stilbenoid, diarylheptanoid and gingerol biosynthesis pathways were significantly upregulated by both SWEs in both crops. This gives evidence for enhanced biosynthesis of stilbenoid, gingerols and diarylheptanoids. Antioxidant, anticancer, antibacterial, anti-inflammatory and antiallergic properties are among the biological and pharmacological effects of these substances (Jiang *et al.* 2017).

The caffeoyl-CoA *O*-methyltransferase was also upregula­ted by the SWEs. *O*-methyltransferases comprise a large group of enzymes that methylate the oxygen atom in a series of secondary metabolites that include flavonoids, alkaloids and phenylpropanoids. This type of methylation is critical in abiotic stress tolerance, disease resistance and even lignin biosynthesis in plants (Bureau *et al.* 2007).

The upregulation of acylsugar coding genes by the SWEs was significant in the current study. Acylsugars belong to a group of specially made plant metabolites that deter pests whereby these compounds are secreted through the trichomes of solanaceous plants (Moghe *et al.* 2017). Furthermore, acylsugars from other species have also been documented to be toxic deterrents to a broad spectrum of herbivores and phytopathogens (Hare 2005; Luu *et al.* 2017).

### Transporters

The transportation mechanism in plants is of paramount importance since it involves several processes including the acquisition of nutrients, developmental processes, cellular homeostasis, as well as for proper communication and for evoking coordinated responses (Larsen *et al.* 2017). In this study, several transporters were upregulated due to the application of SWEs. This coupled with the increase in plant nutrient contents in plants can pave some way to the growth-promoting and disease resistance inducible effects by SWEs. One important group included the highly upregulated nitrate transporters, e.g. high-affinity nitrate transporters (*NRT*). These nitrate transporters have been linked to an array of functions that are all important for plant development including nutrient uptake and translocation, root architecture, storage of vacuole nitrates and proteins, ionic balance, cellular pH, circadian clock-regulated nitrogen and carbon equilibrium, and abiotic and biotic sensing (Fan *et al.* 2017). A study on an *NRT2* gene showed that its overexpression led to overall biomass and yield increase in tomato (Fu *et al.* 2015). *NRT* was also linked to changes in ROS which decreased the infection levels of *Erwinia* in *Arabidopsis thaliana* (Dechorgnat *et al.* 2012).

A transporter enzyme, glutathione *S*-transferase, was also highly expressed in SWE-treated plants. This ubiquitous enzyme has been linked to many processes including the extracellular transport of *trans*-resveratrols and auxin in grapevine (Martínez-Márquez *et al.* 2017). Aquaporin genes were also significantly upregulated in SWE-treated plants. These form membrane channel proteins which are present in all branches of life, but plants have the most diversity in aquaporin homologues. Approximately 47 aquaporins have been identified in tomato and around 73 in sweet pepper (Reuscher *et al.* 2013; Lee *et al.* 2021). They possess key roles in the transportation of small neutral solutes, metal ions and gases as well as being a critical regulator in plant–water relations. Additionally, aquaporins can facilitate the transport of hydrogen peroxide across the membrane, thus contributing to the initiation of plant defence via PAMP-triggered immunity induction and SAR, which are followed by MAPK cascades, callose production, stomatal regulation and the production of PR proteins (Li *et al.* 2020).

The SWEs also activated an ATP-binding cassette as observed in the current study, but only in AS-treated tomato plants. Genes in this cassette regulate the transport of organic acids, phytohormones, metal ions, secondary metabolites and so play crucial roles in plant development (Ofori *et al.* 2018).

A group of genes coding for several metal ion transporters were also upregulated, e.g. potassium channel SKOR, copper transporter 1, Fe (2+) transport protein, zinc transporter 4 and vacuolar iron transporter 1. These metal transporters are involved in the acquisition and translocation of important nutrients which are required in almost all BP, including photosynthesis, and conferring tolerance to abiotic and biotic stress (González-Guerrero *et al.* 2016). For example, the vacuolar iron transporter 1 has been documented as a regular transporter for iron homeostasis in plants through the transportation of cytoplasmic ferrous ions, leading to an increase in iron content in plants (Kato *et al.* 2019). Both AS and SV SWE-treated tomato and sweet pepper had a significant rise in iron content in the current study. Besides the usage of iron for photosynthesis, studies have shown that increased levels of iron can lead to disease reduction in plants by means of defensive generation of ROS (Fones and Preston 2013) and immune-engaged cell death through ferroptosis mechanisms (Herlihy *et al.* 2020). Possibly, these mechanisms together would have contributed to the increased levels of disease resistance observed in SWE-primed plants.

### Phytohormonal modulation in SWE-treated tomato and sweet pepper

Plants produce several phytohormones which are low-molecular-weight natural products, and they are responsible for all developmental and physiological aspects from germination all the way to senescence, as well as in reaction to biotic/abiotic stimuli. In this study, both AS and SV extracts were profiled for auxins, cytokinins, gibberellins, betaines, strigolactones and brassinosteroids. Cytokinin content dominated the hormones tested in both extracts, which was followed by gibberellins and auxins. However, their action on plants given the very low levels of SWE extract application in plants remains sceptical.

Cytokinin is important to plant growth regulation, ranging from functions such as cell division, axillary bud release, photomorphogenic development initiation and shoot apical meristem development (Werner and Schmülling 2009). Interestingly, cytokinin was the highest accumulated hormone in both SWEs and plant tissues compared to the others.

The cytochrome P450 gene was upregulated in all SWE-treated plants, whereas cytokinin catabolic genes were repressed. This gene is a crucial component in cytokinin synthesis mechanism (Xu *et al.* 2015) and as a result, the elevated levels of cytokinin in SWE-treated plant tissues might be explained in part by this observation. This phenomenon was also documented in *A. nodosum* extract-treated *Arabidopsis* plants (Wally *et al.* 2013a).

The auxin content was also high in tomato and pepper tissues in the present study. Many auxin-synthesis-related genes were expressed such as auxin-induced protein 15A, small auxin-up protein 58, auxin response factor 5, etc. Moreover, these transcripts were generally more abundant compared to cytokinin biosynthesis-related genes. The increase in auxin-related genes noted in this study is quite opposite from a previous study on *A. nodosum* extracts on *Arabidopsis.* These contrasting results may be due to differences in the SWE composition as well as variations in plant processes and differences in environmental conditions used for screening plants (Wally *et al.* 2013b).

In the current investigation, the gibberellin concentration in SWE-primed plant tissues was significantly greater in comparison to control plants. This was coupled with the upregulation of genes such as *GA20*-oxidase (*GA20ox*) and *GA3*-oxidase (*GA3ox*) which code for metabolic enzymes in the gibberellin synthesis pathway (Chen *et al.* 2016). Gibberellins are engaged in multiple plant developmental processes encompassing, seed germination, stem extension, expansion of leaves, development of trichomes, maturation of pollen, flowering induction and fruit quality (Achard and Genschik 2009; Chen *et al.* 2016) and many of these effects were previously noted in plants treated with SWEs (Ali *et al.* 2021a; Dookie *et al.* 2021).

Though hormonal analysis of the extracts revealed the presence of phytohormonal substances especially cytokinins, auxins and gibberellins, however, because the rate of application (5 mL L^−1^) to plants which equates to a 200× dilution, the actual available levels of hormones are too low to work on the plant system on their own. Therefore, the hormonal effect seen might be mostly due to the upregulated expression of the hormonal pathway genes that are involved in the synthesis of hormones or downregulation of hormonal catabolism genes which would have eventually caused the observed plant growth stimulation.

## Conclusion

The overall transcriptomic response of both tomato and sweet pepper plants to *A. spicifera* and *S. vulgare* SWEs was the upregulation of key marker genes responsible for both growth stimulation and abiotic and biotic stress tolerance. The study therefore illustrates and warrants further exploration of locally available seaweeds for biostimulant usage in crop production due to their multifaceted effects on crops. The study can be used as a benchmark target for molecular responses in plants due to seaweed biostimulants and to compare the effects observed with other biostimulants and other plant systems. Furthermore, the research bolsters the repertoire for SWE which is a natural organic product to boost sustainability by utilizing this inexpensive bioresource within integrated management approaches rather than capitalizing on a single market niche product.

## Supporting Information

The following additional information is available in the online version of this article—

**Table S1.** Sequencing reads statistics obtained from Novogene Inc. for tomato and sweet pepper.

**Table S2.** Mapping statistics for tomato and sweet pepper using the HISAT2 mapping tool against the respective reference genomes.

**Table S3.** Primer sequence details used for validation of RNA sequencing via qPCR for tomato: (A) AS; (B) SV.

**Table S4.** Primer sequence details used for validation of RNA sequencing via qPCR for sweet pepper: (A) AS; (B) SV.

**Table S5.** Endogenous phytohormone content in sweet pepper and tomato leaves treated with seaweed extracts that were insignificant compared to the control-treated plants.

**Figure S1.** Data processing pipeline for reference-based transcriptomic raw reads.

**Figure S2.** Data processing pipeline for *de novo*-based transcriptomic raw reads.

**Figure S3.** Phred mapping quality score plot output from a representative sample.

**Figure S4.** Blast hit distribution of sequences for tomato (A) and sweet pepper (B).

**Figure S5.** Enzyme hit distribution for sweet pepper and tomato.

plac046_suppl_Supplementary_Material

## Data Availability

The RNA-sequencing fastq data files were deposited in the NCBI’s Short Read Archive (SRA) database under the bioproject PRJNA782171. All the sequence analysis data generated during this study were included in this published article and the **Supporting Information** file.

## Notes

**Phenome, Genome & Environment. Chief Editor**: Colleen Doherty
