# Supplementary material for: Transcriptome-wide modulation by Sargassum vulgare and Acanthophora spicifera extracts results in a prime-triggered plant signalling cascade in tomato and sweet pepper
Source: AoB Plants. 2022 Oct 6;14(6):plac046. doi: 10.1093/aobpla/plac046 (PMC9724562; doi:10.1093/aobpla/plac046)
Supplement: plac046_suppl_Supplementary_Material [file plac046_suppl_supplementary_material.docx]

**Supporting Information**

**Figure S1**. Data Processing pipeline for reference-based transcriptomic raw reads

Expression plots

GO Enrichment

Pathway analysis

DeNovo Assembly- Trinity

Longest ORFs- TransDecoder

Annotation -Blastp

Abundance Estimation- RSEM

Differential Expression- edgeR

**Figure S2**. Data Processing pipeline for de-novo based transcriptomic raw reads

**Table S1**. Sequencing reads statistics obtained from Novogene Inc. for tomato and sweet pepper

| Crop |  | Samples | Raw Reads | Clean Reads | Raw Base (G) | Clean Base (G) | Effective Rate (%) | Error Rate (%) | Q20 (%) | Q30 (%) | GC Content (%) |
| --- | --- | --- | --- | --- | --- | --- | --- | --- | --- | --- | --- |
| Tomato |  | SV rep1 | 24981907 | 24652418 | 7.5 | 7.4 | 98.68 | 0.03 | 97.79 | 93.69 | 43.73 |
|  |  | SV rep2 | 25223949 | 24910039 | 7.6 | 7.5 | 98.76 | 0.03 | 97.73 | 93.56 | 43.84 |
|  |  | AS rep1 | 27466289 | 27005293 | 8.2 | 8.1 | 98.32 | 0.03 | 97.78 | 93.69 | 44.49 |
|  |  | AS rep2 | 28626599 | 28354415 | 8.6 | 8.5 | 99.05 | 0.03 | 97.81 | 93.72 | 44.39 |
|  |  | Control rep1 | 29038200 | 28719736 | 8.7 | 8.6 | 98.9 | 0.03 | 97.78 | 93.68 | 43.91 |
|  |  | Control rep2 | 26110321 | 25838068 | 7.8 | 7.8 | 98.96 | 0.03 | 97.83 | 93.77 | 43.96 |
| Sweet pepper |  | SV rep1 | 31956897 | 31609916 | 9.6 | 9.5 | 98.91 | 0.03 | 97.43 | 93.09 | 43.78 |
|  |  | SV rep2 | 26157576 | 25860520 | 7.8 | 7.8 | 98.86 | 0.03 | 97.57 | 93.42 | 43.78 |
|  |  | AS rep1 | 29254650 | 28918722 | 8.8 | 8.7 | 98.85 | 0.03 | 97.39 | 93.03 | 43.92 |
|  |  | AS rep2 | 26235301 | 25939263 | 7.9 | 7.8 | 98.87 | 0.03 | 97.34 | 92.9 | 44.02 |
|  |  | Control rep1 | 29502038 | 29208588 | 8.9 | 8.8 | 99.01 | 0.03 | 97.53 | 93.35 | 43.67 |
|  |  | Control rep2 | 26095011 | 25816854 | 7.8 | 7.7 | 98.93 | 0.03 | 97.56 | 93.41 | 43.8 |

**Table S2**. Mapping statistics for tomato and sweet pepper using the HISAT2 mapping tool against the respective reference genomes

|  | % Alignment | | % Concordance | | % Discordance | |
| --- | --- | --- | --- | --- | --- | --- |
| Treatment | S. Pepper | Tomato | S. Pepper | Tomato | S. Pepper | Tomato |
| Control1 | 87.83 | 95.00 | 78.88 | 89.28 | 0.68 | 0.61 |
| Control2 | 85.78 | 95.00 | 76.41 | 88.91 | 0.44 | 0.63 |
| AS1 | 91.13 | 93.75 | 82.12 | 87.63 | 0.58 | 0.71 |
| AS2 | 90.46 | 94.42 | 81.26 | 88.65 | 0.58 | 0.58 |
| SV1 | 88.35 | 94.94 | 79.25 | 89.90 | 0.59 | 0.63 |
| SV2 | 88.82 | 94.81 | 79.99 | 89.46 | 0.62 | 0.67 |


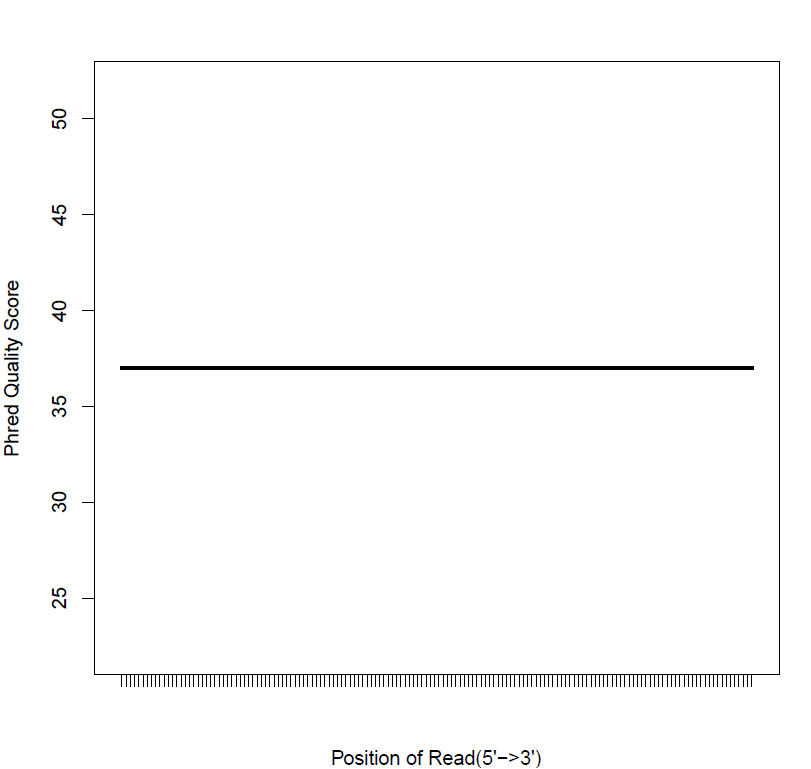


**Figure S3**. Phred mapping quality score plot output from a representative sample

**Figure S4**. Blast Hit distribution of sequences for tomato (A) and sweet pepper (B)


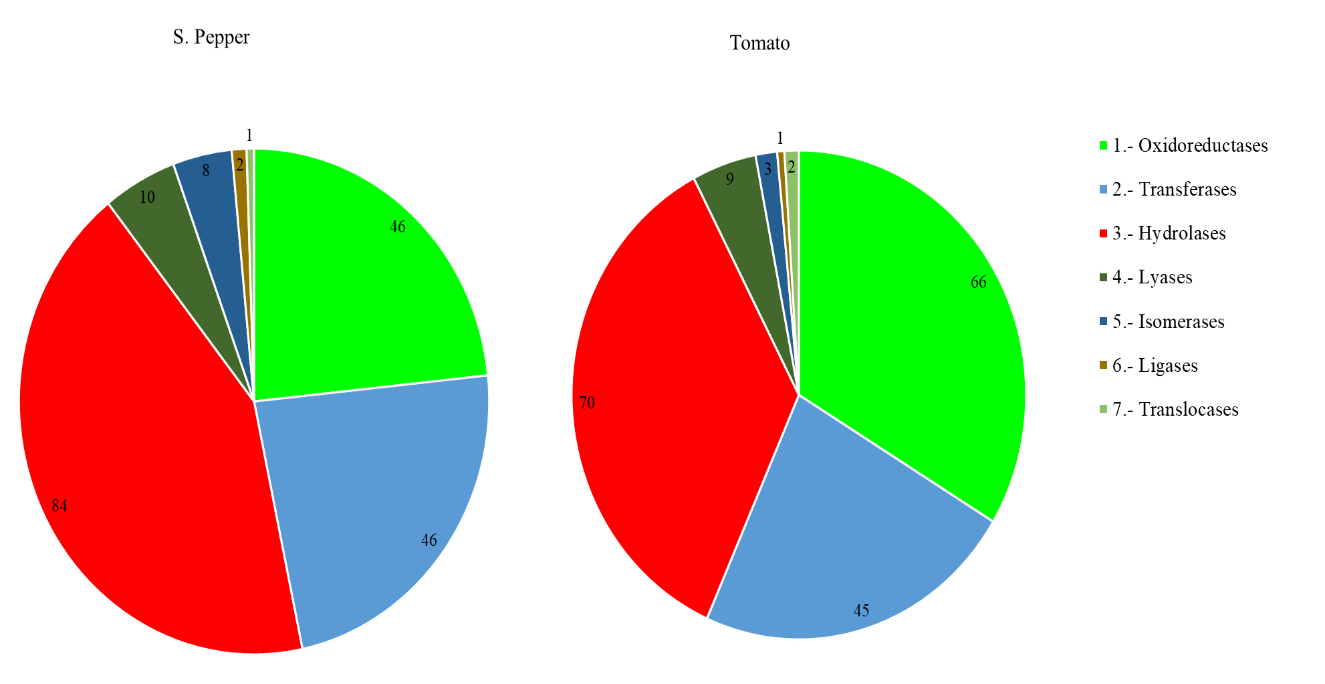


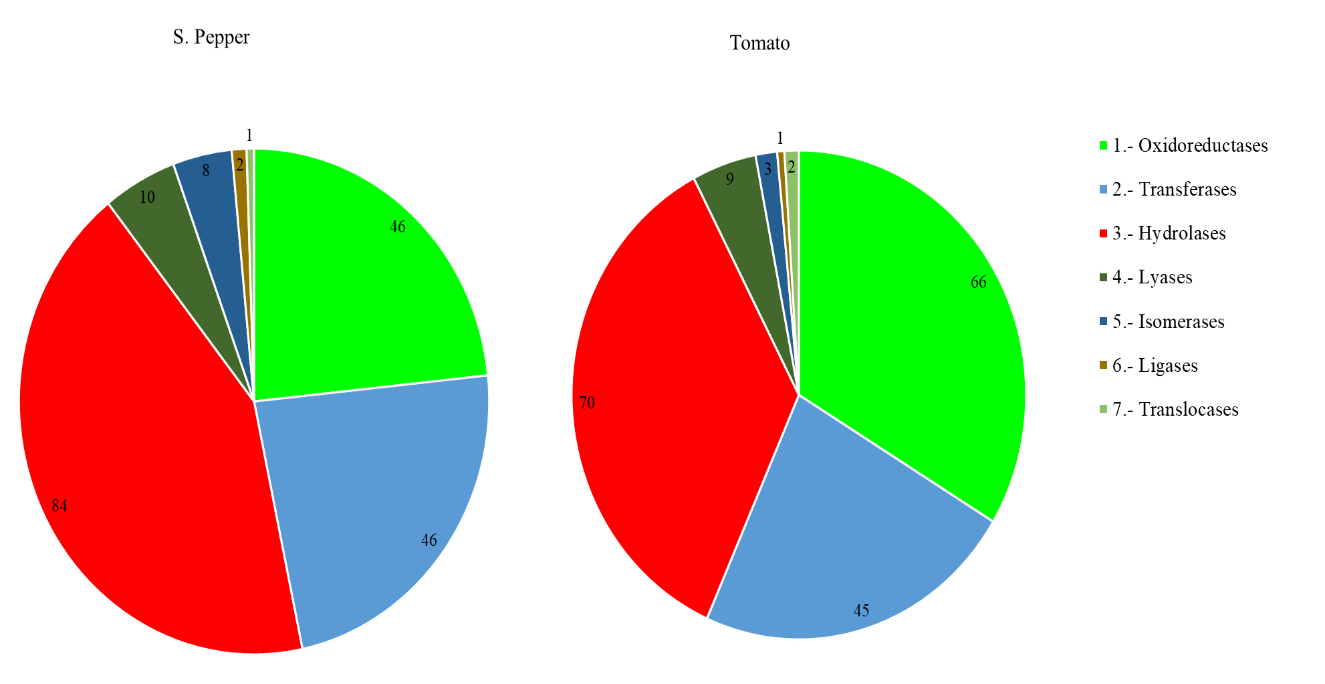


**Figure S5**. Enzyme hit distribution for sweet pepper and tomato

**Table S3**. Primer sequence details used for validation of RNA sequencing via qPCR for tomato: A- AS, B- SV

A

B

**Table S4**. Primer sequence details used for validation of RNA sequencing via qPCR for sweet pepper: A- AS, B- SV

A

B

**Table S5**. Endogenous phytohormone content in sweet pepper and tomato leaves treated with seaweed extracts that were insignificant compared to the control plants.

| Hormone | Sweet Pepper | | | Tomato | | |
| --- | --- | --- | --- | --- | --- | --- |
| Phytohormone | SV | AS | Control | SV | AS | Control |
| Betaines | 0.012 | 0.021 | 0.011 | 0.013 | 0.014 | 0.011 |
| Strigolactones | 0.012 | 0.025 | 0.011 | 0.005 | 0.008 | 0.002 |
| Brassionsteriods | <0.002 | 0.003 | <0.002 | <0.002 | 0.004 | <0.002 |

Data represent the mean of 3 replicate plants per treatment where SD for all samples were less than 0.001. The significance was tested by One-Way ANOVA where P > 0.05.
